# Subliminal stimuli modulate somatosensory perception rhythmically and provide evidence for discrete perception

**DOI:** 10.1038/srep43937

**Published:** 2017-03-09

**Authors:** Thomas J. Baumgarten, Sara Königs, Alfons Schnitzler, Joachim Lange

**Affiliations:** 1 Institute of Clinical Neuroscience and Medical Psychology, Medical Faculty, Heinrich-Heine-University, Düsseldorf 40225, Germany; 2 Department of Experimental Psychology, Faculty of Mathematics and Natural Sciences, Heinrich-Heine-University, Düsseldorf 40225, Germany

## Abstract

Despite being experienced as continuous, there is an ongoing debate if perception is an intrinsically discrete process, with incoming sensory information treated as a succession of single perceptual cycles. Here, we provide causal evidence that somatosensory perception is composed of discrete perceptual cycles. We used in humans an electrotactile temporal discrimination task preceded by a subliminal (i.e., below perceptual threshold) stimulus. Although not consciously perceived, subliminal stimuli are known to elicit neuronal activity in early sensory areas and modulate the phase of ongoing neuronal oscillations. We hypothesized that the subliminal stimulus indirectly, but systematically modulates the ongoing oscillatory phase in S1, thereby rhythmically shaping perception. The present results confirm that, without being consciously perceived, the subliminal stimulus critically influenced perception in the discrimination task. Importantly, perception was modulated rhythmically, in cycles corresponding to the beta-band (13–18 Hz). This can be compellingly explained by a model of discrete perceptual cycles.

Although perception appears smooth and continuous in our subjective experience, it has been discussed whether the nature of sensory information processing is intrinsically discrete. Within such a framework, incoming sensory information would be grouped in consecutive separated perceptual cycles or snapshots^1^,^2^,^3^. A snapshot or perceptual cycle, thus, forms the temporal unit of perceptual experience, leading to rhythmic or cyclic perception^4^. While the ongoing debate whether perception is continuous or discrete has been put forward at least a century ago^5^,^6^, the hypothesis of discrete perception has only recently regained new support from neuroimaging studies. These studies have shown that the periodic modulation of subjects’ perception was related to the phase of ongoing neuronal oscillations in the alpha and beta band located in the parieto-occipital or primary somatosensory cortex (S1)^7^,^8^,^9^. Said neuronal oscillations might thus form the neurophysiological basis of periodic modulations of perception, suggesting that neuronal oscillations in specific frequencies define perceptual cycles. However, current experimental evidence for discrete perception and its putative underlying neuronal mechanisms is mostly of correlative nature, while causal evidence remains scarce^10^,^11^. Consequently, the theory of discrete perception remains controversially discussed^12^,^13^. To advance this discussion, it would be necessary to causally modulate the rhythmic patterns of perception (i.e., the perceptual cycles). Here, we use the term causal to define a process in which an independent variable (e.g., the onset of a putative perceptual cycle on behavioral level or the phase of neuronal oscillations on neurophysiological level) is experimentally and systematically modulated while measuring the corresponding changes on the dependent variable (i.e., rhythmic perception). This causal approach stands in contrast to the simultaneous measurement of both variables without systematic variation, which would result in correlative evidence. The causal approach would allow for the possibility to gather experimental evidence for or against the theory of discrete perception and shed light on the patterns of perceptual cycles. We assessed this relationship by using an electrotactile temporal discrimination task which was preceded by a subliminal (i.e., below perceptual threshold) stimulus. Operationally, the use of subliminal stimuli is advantageous compared to the use of suprathreshold stimuli. Because subliminal stimuli intensities are insufficient to initiate global network activity^14^,^15^ and these stimuli are not consciously perceived, the risk of perceptually confusing preceding subliminal stimuli with the subsequent target stimuli of the temporal discrimination task or masking the target stimuli is minimized. In addition, suprathreshold stimuli might attract exogenous (i.e., task-independent) and conscious attention. Conscious attention has been utilized in previous studies to induce a reset event. These studies have shown that conscious attention can trigger rhythmical patterns of behavior and neuronal activity^7^,^16^,^17^, which might interfere with the proposed cycles of perception. While we cannot exclude that a subliminal stimulus also triggers (unconscious) attentional mechanisms, the subliminal stimulus enables us to exclude conscious attention mechanisms and investigate how an unconsciously perceived event modulates perception. Despite not being consciously perceived, subliminal stimuli trigger neuronal activity in early sensory areas^15^,^18^,^19^. Other studies report subliminal stimuli to elicit weak evoked responses in somatosensory areas^19^,^20^ and fMRI BOLD decreases related to functional inhibition^19^,^21^. Albeit not being consciously perceived, subliminal stimuli have been shown to affect the perception of subsequently presented stimuli^22^. This effect on perception is presumably mediated by the modulation of phase of ongoing neuronal oscillations in sensory areas (e.g., refs 23, 24). This process of phase resetting is well documented within and across sensory modalities for suprathreshold stimuli^25^,^26^,^27^,^28^, whereas reports on subthreshold stimuli remain scarce^20^. We hypothesized that by presenting the subliminal stimulus at systematically varying time points relative to the discrimination task, the phase of ongoing neuronal oscillations and consequently the starting point of a perceptual cycle would be modulated systematically (though indirectly; see ref. 24 for a similar paradigm in the visual domain). Accordingly, perception in the discrimination task should vary rhythmically. Such results would provide valuable evidence for discrete perception which would go beyond studies that report correlative evidence for perceptual cycles.

## Results

**Figure 1:**
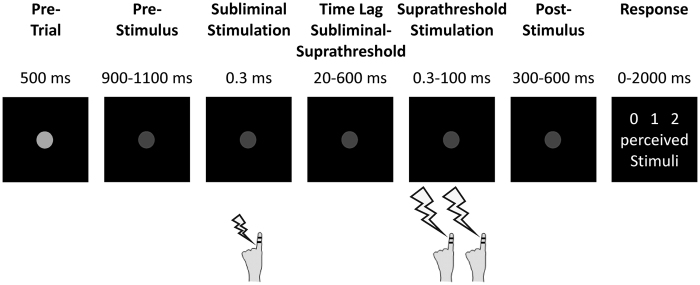
Experimental setup. Subjects fixated a central grey dot. After a jittered pre-stimulus period, they received one subliminal electrotactile stimulus (i.e., below perceptual threshold) on their left index finger, followed by a time lag (20–600 ms) in which only the fixation dot was present. Then subjects received two suprathreshold electrotactile stimuli with varying SOA. After another jittered time period (300–600 ms), written instructions prompted the subjects to report their perception.

Subjects performed a temporal perceptual discrimination task (see Materials and Methods section for details) in which they received either zero, one or two suprathreshold electrotactile target stimuli separated by specific stimulus onset asynchronies (SOAs; Fig. 1)^9^,^29^. Crucially, these target stimuli were preceded by a subliminal electrotactile stimulus. The time lag between the subliminal stimulus and the first target stimulus was systematically varied (20–600 ms). After presentation of the target stimuli, subjects had to report the amount of perceived electrotactile stimuli (i.e., zero, one, or two stimuli).

**Figure 2:**
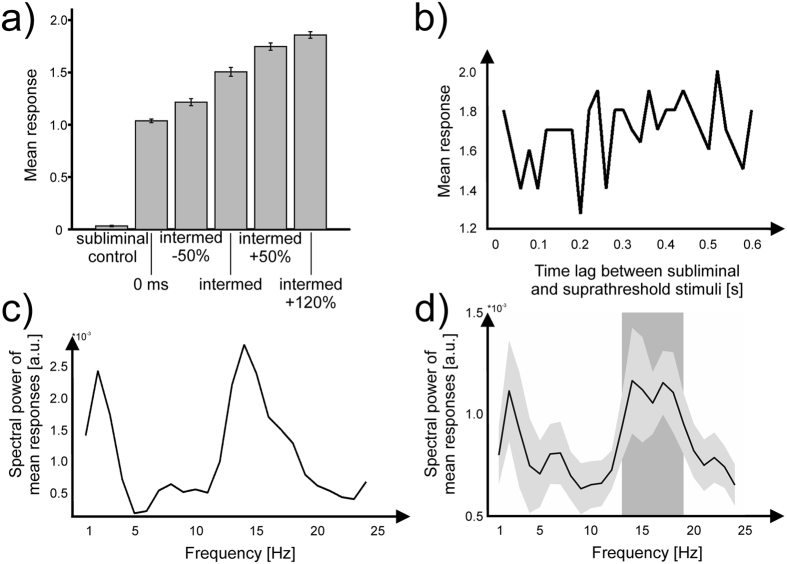
Behavioral data. (**a**) Average number of perceived suprathreshold stimuli displayed separately for all conditions (i.e., different SOAs) averaged across all subjects and time lags between subliminal and suprathreshold stimuli. Data are presented as mean ± SEM. (**b**) Exemplary single subject average number of perceived suprathreshold stimuli of the intermed-condition as a function of time lag between subliminal and suprathreshold stimuli. t = 0 denotes the onset of the subliminal stimulus. Time points indicate the time lag between subliminal stimulus and first suprathreshold target stimulus. (**c**) Spectral decomposition of the exemplary single subject data in (**b**). (**d**) Same as (**c**), but now averaged across all subjects. The shaded box highlights frequencies with significantly increased amplitudes (p = 0.002, corrected for multiple comparisons). The grey shading represents the SEM.

When no target stimuli were presented, but only the subliminal stimulus (i.e., the control condition), subjects on average perceived 0.03 ± 0.03 stimuli [mean ± SD] (Fig. 2A), demonstrating that the subliminal stimuli were not perceived as target stimuli. After presentation of one target stimulus, subjects perceived 1.04 ± 0.08 stimuli, averaged across all time lags between subliminal and target stimuli. When two target stimuli were presented, subjects’ responses increased monotonically with increasing SOA between the two stimuli (Fig. 2A; see Materials and Methods section for details). A repeated measures ANOVA revealed highly significantly different responses across conditions (F(5,95) = 666.5, p < 0.01). Post-hoc pairwise t-tests revealed highly significant differences between all SOAs (p < 0.01 for all comparisons).

Next, we investigated potential periodic relationships between subjects’ response rates and the time lag between subliminal stimulus and target stimuli by applying Fourier transformation on perceptual response rates (see Fig. 2B,C for exemplary single subject data, see Supplementary Figure 1 for an overview of all single subject data; see Fig. 2D for the group-level average data). The spectra showed a highly significant peak between 13–18 Hz (p < 0.01) and a second peak between 1–2 Hz which, however, did not reach statistical significance (p = 0.11; Fig. 2D). To assess whether the rhythmic modulation of perception was phase-locked, i.e., whether the subliminal stimulus induced a phase resetting, we computed the average phase angle across the entire interval between subliminal stimulus and the first target stimulus for each subject. For each subject, we selected the phase angle of the specific frequency within the beta-band (13–24 Hz) showing the highest amplitude. The selected phase angles (−1.92 ± 1.03 radians [mean ± SD]) significantly differed from a uniform distribution (Rayleigh test for non-uniformity across subjects; z = 4.485, p < 0.01). In addition, phase consistency computed for each frequency separately (i.e., without a-priori selection of the individual frequency) across subjects showed a peak of phase consistency between ~12–16 Hz, which however did not differ significantly from a uniform distribution.

## Discussion

We investigated if perception in an electrotactile temporal discrimination task is influenced by a preceding subliminal stimulus. Despite not being perceived consciously, the subliminal stimulus modulated perception rhythmically with a periodicity of 13–18 Hz. Furthermore, phase angles within the beta-band across subjects significantly differed from a uniform distribution, indicating consistent phase across subjects.

**Figure 3:**
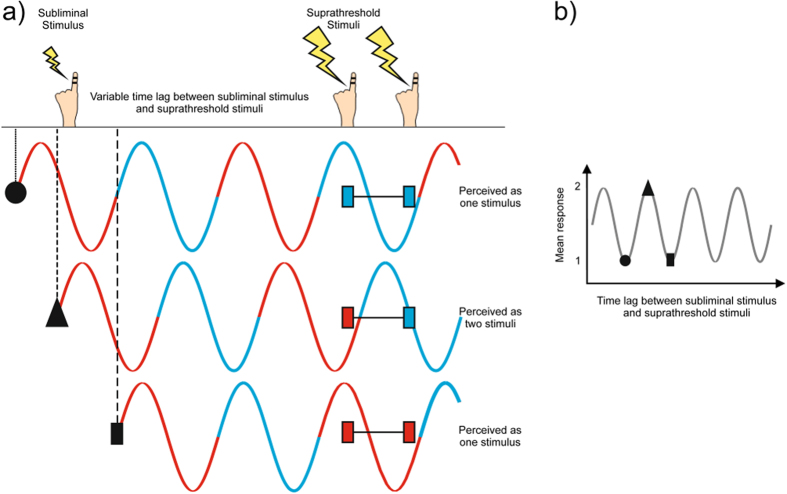
Model for perceptual cycles. (**a**) Three illustrative trials with different time lags between subliminal stimulus and suprathreshold stimuli. Each subliminal stimulus resets neuronal oscillations (indicated by black circle, triangle and rectangle). Perceptual cycles in neuronal oscillations are represented by red and blue lines. Different time lags result in suprathreshold stimuli falling in either one (black circle and rectangle) or two (black triangle) perceptual cycles, which results in perception of either one stimulus (black circle and rectangle) or two separate stimuli (black triangle). (**b**) Schematic representation of periodic relation between number of perceived suprathreshold stimuli and the time lag between subliminal stimulus and suprathreshold stimuli as a result of the model in (**a**).

We propose an explanation of the results based on our recent findings in an MEG study^9^. Here, subjects received two electrotactile stimuli (similar to the present study, but without any subliminal stimuli). Subjects’ perception varied between one or two perceived stimuli from trial to trial. Perceptual variability depended on the phase of ongoing neuronal oscillations in the alpha and lower beta frequency band (8–20 Hz) in S1. We have proposed a model stating that if the two electrotactile stimuli fall within one cycle of the 8–20 Hz oscillations, they are perceived as a single stimulus, but if they fall within separate cycles, they are perceived as two distinct stimuli (Fig. 3). Accordingly, our model states that cycles of neuronal oscillations in the alpha-/beta-band define discrete perceptual cycles in the somatosensory domain. We propose that this model explains the present results: In ongoing neuronal oscillations, the phases - and thus the perceptual cycles - are randomly distributed with respect to the to-be-perceived target stimuli. Consequently, also subjects’ perception varies randomly from trial to trial (provided that the SOA is smaller than the cycle length). In the present study, the subliminal stimulus presumably resets the phase of ongoing neuronal oscillations^20^, which is supported by the finding of consistent phase across subjects within the beta-band. Depending on the time-lag between subliminal stimulus and the first target stimulus, this phase reset determines if the two target stimuli fall within one or two cycles, leading to the perception of one or two stimuli, respectively (Fig. 3). Accordingly, the frequency of the rhythmic variation in perception is determined by the cycle length of those neuronal oscillations that define the discrete perceptual cycles. Based on the MEG data, we proposed that the perceptual cycles are defined by neuronal oscillations in the 8–20 Hz frequency band^9^. This proposition is confirmed by the 13–18 Hz fluctuation of perception induced by the subliminal stimulus (Fig. 2D). However, since the present effect is located at the lower end of the classical beta band and in between the “classical” centers of somatosensory alpha (~10 Hz) and beta (~20 Hz) oscillations (or mu-rhythm^30^,^31^), a clear distinction from the alpha frequency band remains difficult. Accordingly, in our previous MEG study, we found a significant phase difference between perceiving “2” and “1” stimuli in the frequency range 8–20 Hz, thus encompassing the classical alpha- as well as the lower beta-band (albeit more strongly pronounced in the beta-band^9^). Although the behavioral effects in our present study do not show a peak in the classical alpha-band (8–12 Hz), it still remains not fully clear which frequency band(s) the effect might be assigned to. Future MEG/EEG studies investigating the neurophysiological basis of a phase resetting, might clarify this question.

Most previous studies providing evidence for a causal influence of neuronal oscillations on perception modulated neuronal oscillations by inducing an external rhythm to the brain^10^,^11^,^25^. In contrast, we do not induce an external rhythm to the brain nor does our single subliminal stimulus contain a temporal structure. Thus, any rhythmicity in the data cannot be explained by an externally induced rhythm but is putatively due to reset of ongoing neuronal oscillations^16^.

Recent studies reported rhythmic modulations of behavioral performance following within-modality or crossmodal reset stimuli^16^,^26^. While these studies investigated visual perception, our results provide novel evidence for rhythmic patterns of somatosensory perception. Furthermore, these studies often found low frequency rhythms in the delta to alpha range (<1 to 12 Hz) and assigned the rhythmic pattern to rhythmic fluctuations of visual attention^16^. In contrast, we find the significant rhythmic fluctuations in the beta-band in the somatosensory domain. Most importantly, these studies did not address the question of whether perception is a continuous or discrete process. In addition to the few studies providing evidence for discrete perceptual cycles in the visual and somatosensory domain, our results critically extend these studies by demonstrating that perception can be systematically modulated as predicted by a model of perceptual cycles^9^.

Subliminal stimulation intensities were selected for the preceding stimulus in order to guarantee that performance in the temporal discrimination task relied solely on the suprathreshold target stimuli (i.e., that the preceding stimulus would not be perceptually confused with the target stimuli). One might expect that a suprathreshold preceding stimulus would likewise, or even more likely, elicit a phase reset and thus lead to similar results. A suprathreshold stimulus might, however, additionally trigger conscious attentional sampling mechanisms^7^,^16^,^17^. Such conscious attentional sampling mechanisms might interfere with our proposed perceptual cycles. While we cannot exclude that the subliminal stimulus also triggers (unconscious) attentional mechanisms, we were able to study a phase reset that is unnoticed by subjects. In addition, a preceding suprathreshold stimulus could perceptually mask the subsequent target stimuli. This would affect the perception of the target stimuli and could even render the target stimuli near invisible for short intervals between preceding and target stimuli (see ref. 16 for a similar effect). Thus, by using a subliminal stimulus, the results are less likely confounded by other, unintentional processes. In addition, we believe that our results are even more intriguing due to the fact that subliminal stimulation can modulate perception.

In line with studies demonstrating phase resets in response to suprathreshold stimuli (e.g., refs 26, 27, 28), there is evidence that subliminal tactile stimuli can induce oscillatory phase resets in the somatosensory cortex^20^ and the finding of consistent phase across subjects likewise suggests such a phase reset. Nonetheless, it should be noted that our behavioral approach provides no direct measure of oscillatory phase. Thus, although the theory of phase resets has been brought forward by other studies (e.g., refs 23, 24) and is compelling and offers an elegant explanation for our results, future MEG/EEG studies should aim to confirm the present hypothesis of a phase reset of neuronal oscillations as the underlying process of our results. To conclude, our findings demonstrate a rhythmic modulation in the beta-band (13–18 Hz) of perception by subliminal, i.e., not consciously perceived stimuli. The findings support a model of perceptual cycles in the somatosensory domain^9^. The results provide novel causal evidence for discrete and cyclic perception.

## Materials and Methods

### Subjects

Twenty-five healthy subjects participated in the study after providing written informed consent in accordance with the Declaration of Helsinki. The study and methods were approved by the Ethical Committee of the Medical Faculty, Heinrich-Heine-University Düsseldorf, and in line with the guidelines of the Declaration of Helsinki. All subjects had normal or corrected-to-normal vision and reported no sensory impairments, known history of neurological disorders or use of neuro-modulatory medication. Two subjects had to be excluded because they perceived the subliminal stimulation even at minimal stimulation amplitude. Three subjects (#4, #9, #22) were excluded from further analysis because they either perceived subliminal stimulation or because they showed a bottom or ceiling effects in their response distribution (see Analysis section for a detailed explanation of the exclusion criteria). Thus, twenty subjects (13 females, age: 27.6 ± 5.6 years [mean ± SD]) remained for further analysis.

### Stimuli and Procedure

Subjects were seated in a dimmed and sound-attenuated room. Visual instructions were projected on a translucent screen (60 Hz refresh rate), which was centrally positioned 57 cm in front of the subjects. Each trial started with the presentation of a light grey dot in the middle of the screen for 500 ms (Fig. 1). Next, the light grey dot decreased in luminance, signaling the start of the stimulation period. After a jittered time period of 900–1100 ms in which only the fixation dot was present, subjects received electrotactile stimuli on their left index finger. First, subjects were stimulated with one subliminal stimulus (i.e., stimulation with subthreshold amplitude levels) followed by zero, one, or two suprathreshold target stimuli (see below for details on stimulation parameters). The subliminal stimulus was applied by means of an electrode pair located at the base of the left index finger. Current amplitudes of the subliminal stimulation (1.2 ± 0.3 mA [mean ± SD]) were determined individually for each subject prior to the experiment and set to 85% of the individual perceptual threshold, so that subjects did not consciously perceive this stimulus. Target stimuli were applied by means of an electrode pair located at the tip of the left index finger. Target stimuli amplitudes (2.5 ± 0.5 mA) were individually set to a level where subjects could clearly perceive stimulation, but below pain threshold. The time lag between the subliminal stimulus and the first target stimulus were pseudo-randomly varied from 20 to 600 ms in steps of 20 ms. All electrotactile stimuli were applied for 0.3 ms and generated by a Stimulus Current Generator (DeMeTec GmbH, Langgöns, Germany). After stimulation, the fixation dot was present for a jittered time period between 300–600 ms before written instructions were presented. Subjects had to report their perception of the target stimuli, i.e., if they perceived either zero, one single or two temporally separate stimuli. If subjects did not respond within 2 seconds or responded before the presentation of the instructions, a warning was presented visually and the trial was repeated at the end of the block. Responses were given by button press with the index, middle and ring finger of the right hand. Button configurations for reporting one or two stimuli were randomized from trial to trial between the right index and middle finger. The perception of zero stimuli was always reported by a button press with the right ring finger. No further feedback was given.

Prior to each experiment, we presented to each subject suprathreshold target stimuli (without subliminal stimuli) with varying stimulus onset asynchronies (SOA). This way, we determined in a staircase procedure the individual SOA for which the respective subject perceived stimulation with two suprathreshold electrical stimuli as two separate stimuli in 50% of all trials and as one stimulus in the other 50% of trials (subsequently labeled intermediate SOA; 31.9 ± 15.7 ms; average difference 8.2 ± 15.1 ms (mean ± SD) across blocks). In the following main experiment, subjects were stimulated with two target stimuli separated by this intermediate SOA in 300 trials. In addition, subjects were stimulated with two target stimuli separated by an SOA with ±50% length of the intermediate SOA in 90 trials, respectively. Furthermore, trials with a predetermined SOA of 0 ms (i.e., only one stimulus was presented) and trials with long SOA (+120% intermediate SOA length) were presented in 60 trials, respectively. Finally, in on average 60 trials no target stimuli were presented. This condition served as a control condition (subsequently labeled subliminal control) to guarantee that the subjects did not perceive the subliminal stimulation. In summary, subjects received 660 trials presented in randomized order.

The experiment consisted of two identical blocks. Each block began with the staircase procedure in order to determine the individual intermediate SOA, followed by the main experiment containing 660 trials as described above. After 200 trials, subjects had the possibility to take self-paced breaks. In addition, subjects were offered a break between the two blocks. Each block had a duration of ~20 min.

Stimulus presentation was controlled by means of Presentation software (Neurobehavioral Systems, Albany, NY, USA). Before beginning the experiment, each subject received instructions of the experimental task but remained naïve to the purpose of the experiment.

### Analysis

Behavioral data were first analyzed with regard to perceptual response rates (i.e., perceived zero, one or two stimuli) for each condition (subliminal control, 0 ms SOA, intermediate SOA, ± 50% intermediate SOA, and 120% intermediate SOA), pooled across all time lags between subliminal and target stimuli. Perceptual response rates were averaged across both blocks and across subjects and compared across conditions by means of a repeated measures ANOVA and post-hoc paired t-tests. For the analysis of perceptual response rates as a function of the time lag between subliminal stimulation and the first target stimulus, only trials with intermediate SOA were analyzed. All other conditions (subliminal control, 0 ms SOA, ±50% intermediate SOA, and 120% intermediate SOA,) served only as control conditions and/or to mask the main condition in order to minimize learning effects or perceptual biases. Subliminal control trials (i.e., trials in which only the subliminal stimulus was presented, without target stimuli) were used as a control condition to guarantee that subjects did not perceive the subliminal stimulation. Blocks in which subjects reported to perceive >10% of subliminal control trials were discarded from further analysis (6 blocks rejected). Blocks in which response rates showed bottom or ceiling effects (mean perception of either “1” or “2” in two or more adjacent time lags in trials with intermediate SOA) were discarded from analysis, because these bottom or ceiling effects would have affected the spectral decomposition (6 blocks rejected).

For all trials with intermediate SOA, we computed for each block mean response rates for each subject as a function of time lag between subliminal and target stimuli. To this end, individual mean response rates were computed for each 20 ms shift of the subliminal stimulus relative to the target stimuli (i.e., subliminal stimulus presented 600 ms vs. 580 ms vs. 560 ms … vs. 20 ms before the first target stimulus), resulting in a temporal resolution of 20 ms (i.e., 50 Hz, resulting in a Nyquist frequency of 25 Hz). To investigate potential periodic relationships between perceptual response rates and the time lag between subliminal stimulation and the first target stimulus, we computed a Fourier transformation on the perceptual response rates within each block. Perceptual reports were zero padded (1000 ms trial length) and multiplied with a single Hanning taper before Fourier transformation. Spectral analysis was performed for frequencies between 1 and 24 Hz (i.e., below the Nyquist frequency) in steps of 1 Hz. Subsequently, we averaged for each subject the results of the two Fourier transformations (one per block).

Statistical analysis of the spectral amplitudes was performed using a nonparametric randomization approach^32^. The null hypothesis states that perceptual reports are independent of the time lag between subliminal stimulation and target stimuli. Since regarding to the null hypothesis, there is no periodicity or other temporal structure in the perceptual performance, time points are exchangeable. Thus, we randomly exchanged time points 1000 times to generate a randomization distribution against which observed data were compared^16^. These randomizations were performed for each subject individually (i.e., for each subject and for each block separately). For each randomization, we performed the same analysis as for the observed data as described above. This procedure resulted in 1000 spectra for each subject and block, which constituted the null distribution per subject and block. Then, we combined per subject the null distributions of the two blocks to achieve one null distribution per subject for further analysis. Next, we statistically tested for each frequency independently the observed data against the null distribution across subjects by means of a nonparametric permutation approach^16^,^32^. First, we took the median of the null distribution and computed t-values between observed data and the median value by means of an independent t-test. This approach resulted in t-values (not corrected for multiple comparisons) for each frequency. Secondly, we applied a non-parametric cluster-based permutation approach to correct for multiple comparisons^32^. To this end, we thresholded the t-values at t = 1.96 (p < 0.05). This resulted in clusters of adjacent frequencies. Cluster-level test statistics were calculated by taking the sum of the t-values within a cluster. Next, we computed a cluster-level null distribution by re-computing the frequency t-maps after randomly permuting the data (under the null hypothesis of no difference, and thus exchangeability, between observed data and shuffled data). This process of random permutation was repeated 1000 times. For each repetition, we re-computed the cluster-level statistics as described above, which served as the cluster-level null distribution. The proportion of elements in the null distribution exceeding the observed cluster-level test statistic was used to estimate a p-value for each cluster. This statistical approach effectively controls for multiple comparisons across time points and channels (see ref. 32 for a detailed discussion on cluster-based nonparametric tests) and has been used for statistical control of similar behavioral data (e.g., refs 16, 17). This analysis corresponds to a random effects analysis^16^.

Analysis of phase was based on the complex output of the Fourier transformation of the perceptual response rates per block. Fourier transformation parameters were equal to the spectral analysis (see above). For each block of each subject, phase angles were computed for each frequency (1–24 Hz), then normalized by their amplitude and averaged over blocks. For each subject, we determined the frequency showing the highest amplitude within the beta-band range (13–24 Hz) based on the across-block averaged Fourier transformations. Average phase angles for this individual frequency were selected for each subject, respectively, and statistically compared against a uniform distribution by means of a Rayleigh test.

We also computed phase consistency across subjects for all frequencies (i.e., without a-priori selection of the individual frequency). To this end, we computed the complex output of the fast Fourier transformation (FFT) of the perceptual response rates per block (see Material and Methods for parameters of the FFT above). For each block of each subject, phase angles were computed for each frequency (1–24 Hz), then normalized by their amplitude and averaged over blocks. Finally, we averaged the phase angles per frequency across subjects.

All data analysis was performed using Matlab (Mathworks inc., Natick, MA, USA) and the FieldTrip toolbox^33^ (www.fieldtriptoolbox.org). Circular data analysis was performed using the CircStat toolbox^34^.

## Additional Information

**How to cite this article:** Baumgarten, T. J. *et al*. Subliminal stimuli modulate somatosensory perception rhythmically and provide evidence for discrete perception. *Sci. Rep.* **7**, 43937; doi: 10.1038/srep43937 (2017).

**Publisher's note:** Springer Nature remains neutral with regard to jurisdictional claims in published maps and institutional affiliations.

## Supplementary Material

Supplementary Figures

## References

[b1] Stroud J. M. In Information theory in psychology: problems and methods(Free Press, New York, NY, US), pp. 174–207 (1956).

[b2] Harter M. R. Excitability cycles and cortical scanning: A review of two hypotheses of central intermittency in perception. Psychol Bull. 68, 47–58 (1967). 4859873 10.1037/h0024725

[b3] VanRullen R. & Koch C. Is perception discrete or continuous? Trends Cogn Sci. 7, 207–213 (2003). 12757822 10.1016/s1364-6613(03)00095-0

[b4] VanRullen R. Perceptual Cycles. Trends Cogn Sci. 20, 723–735 (2016). 27567317 10.1016/j.tics.2016.07.006

[b5] von Baer K. E. In Aus baltischer Geistesarbeit. Reden und Aufsätze. Vol. 1, edited by Keyserling Alexander (Jonck and Poliewsky, Riga, 1908).

[b6] Bergson H. Creative evolution(Henry Holt, New York, NY, US, 1911).

[b7] Busch N. A. & VanRullen R. Spontaneous EEG oscillations reveal periodic sampling of visual attention. Proc Natl Acad Sci USA 107, 16048–16053 (2010). 20805482 10.1073/pnas.1004801107 PMC2941320

[b8] Chakravarthi R. & VanRullen R. Conscious updating is a rhythmic process. Proc Natl Acad Sci USA 109, 10599–10604 (2012). 22689974 10.1073/pnas.1121622109 PMC3387058

[b9] Baumgarten T. J., Schnitzler A. & Lange J. Beta oscillations define discrete perceptual cycles in the somatosensory domain. Proc Natl Acad Sci USA 112, 12187–12192 (2015). 26324922 10.1073/pnas.1501438112 PMC4593118

[b10] Cecere R., Rees G. & Romei V. Individual differences in alpha frequency drive crossmodal illusory perception. Curr Biol. 25, 231–235 (2015). 25544613 10.1016/j.cub.2014.11.034 PMC4300399

[b11] Gundlach C., Müller M. M., Nierhaus T., Villringer A. & Sehm B. Phasic modulation of human somatosensory perception by transcranially applied oscillating currents. Brain Stimul.(2016). 10.1016/j.brs.2016.04.014 27237962

[b12] Holcombe A. O., Clifford C. W., Eagleman D. M. & Pakarian P. Illusory motion reversal in tune with motion detectors. Trends Cogn Sci. 9, 559–560 (2005). 16271506 10.1016/j.tics.2005.10.009

[b13] Kline K. A. & Eagleman D. M. Evidence against the temporal subsampling account of illusory motion reversal. J Vis. 8, 13 (2008). 10.1167/8.4.13 PMC2856842 18484852

[b14] Dehaene S., Changeux J.-P., Naccache L., Sackur J. & Sergent C. Conscious, preconscious, and subliminal processing: a testable taxonomy. Trends Cogn Sci. 10, 204–211 (2006). 16603406 10.1016/j.tics.2006.03.007

[b15] Weisz N. . Prestimulus oscillatory power and connectivity patterns predispose conscious somatosensory perception. Proc Natl Acad Sci USA 111, E417–E425 (2014). 24474792 10.1073/pnas.1317267111 PMC3910583

[b16] Landau A. N. & Fries P. Attention samples stimuli rhythmically. Curr Biol. 22, 1000–1004 (2012). 22633805 10.1016/j.cub.2012.03.054

[b17] Landau A. N., Schreyer H. M., van Pelt S. & Fries P. Distributed attention is implemented through theta-rhythmic gamma modulation. Curr Biol. 25, 2332–2337 (2015). 26279231 10.1016/j.cub.2015.07.048

[b18] Ress D. & Heeger D. J. Neuronal correlates of perception in early visual cortex. Nat Neurosci. 6, 414–420 (2003). 12627164 10.1038/nn1024 PMC2278238

[b19] Nierhaus T. . Imperceptible somatosensory stimulation alters sensorimotor background rhythm and connectivity. J Neurosci. 35, 5917–5925 (2015). 25878264 10.1523/JNEUROSCI.3806-14.2015 PMC6605170

[b20] Palva S., Linkenkaer-Hansen K., Näätänen R. & Palva J. M. Early neural correlates of conscious somatosensory perception. J Neurosci. 25, 5248–5258 (2005). 15917465 10.1523/JNEUROSCI.0141-05.2005 PMC6724814

[b21] Blankenburg F. . Imperceptible stimuli and sensory processing impediment. Science 299, 1864 (2003). 12649475 10.1126/science.1080806

[b22] Bauer F., Cheadle S. W., Parton A., Müller H. J. & Usher M. Gamma flicker triggers attentional selection without awareness. Proc Natl Acad Sci USA 106, 1666–1671 (2009). 19124766 10.1073/pnas.0810496106 PMC2635817

[b23] Diederich A., Schomburg A., Colonius H. & Hamed S. B. Saccadic reaction times to audiovisual stimuli show effects of oscillatory phase reset. PLoS One 7, e44910 (2012). 23056186 10.1371/journal.pone.0044910 PMC3463580

[b24] Drewes J., Zhu W., Wutz A. & Melcher D. Dense sampling reveals behavioral oscillations in rapid visual categorization. Sci Rep. 5, 16290 (2015). 26542183 10.1038/srep16290 PMC4635344

[b25] Lakatos P., Karmos G., Mehta A. D., Ulbert I. & Schroeder C. E. Entrainment of neuronal oscillations as a mechanism of attentional selection. Science 320, 110–113 (2008). 18388295 10.1126/science.1154735

[b26] Romei V., Gross J. & Thut G. Sounds reset rhythms of visual cortex and corresponding human visual perception. Curr Biol. 22, 807–813 (2012). 22503499 10.1016/j.cub.2012.03.025 PMC3368263

[b27] Fiebelkorn I. C., Saalmann Y. B. & Kastner S. Rhythmic sampling within and between objects despite sustained attention at a cued location. Curr Biol.: CB 23, 10.1016/j.cub.2013.10.063 (2013). PMC3870032 24316204

[b28] Mercier M. R. . Neuro-oscillatory phase alignment drives speeded multisensory response times: an electro-corticographic investigation. J Neurosci. 35, 8546–8557 (2015). 26041921 10.1523/JNEUROSCI.4527-14.2015 PMC6605331

[b29] Baumgarten T. J., Schnitzler A. & Lange J. Prestimulus alpha power influences tactile temporal perceptual discrimination and confidence in decisions. Cereb Cor. 26, 891–903 (2016). 10.1093/cercor/bhu247 25331603

[b30] Salmelin R. & Hari R. Spatiotemporal characteristics of sensorimotor neuromagnetic rhythms related to thumb movement. Neuroscience 60, 537–550 (1994). 8072694 10.1016/0306-4522(94)90263-1

[b31] Salenius S., Portin K., Kajola M., Salmelin R. & Hari R. Cortical control of human motoneuron firing during isometric contraction. J Neurophysiol 77, 3401–3405 (1997). 9212286 10.1152/jn.1997.77.6.3401

[b32] Maris E. & Oostenveld R. Nonparametric statistical testing of EEG- and MEG-data. J Neurosci Methods. 164, 177–190 (2007). 17517438 10.1016/j.jneumeth.2007.03.024

[b33] Oostenveld R., Fries P., Maris E. & Schoffelen J.-M. FieldTrip: Open source software for advanced analysis of MEG, EEG, and invasive electrophysiological data. Comput Intell Neurosci 2011, 156869 (2010). 21253357 10.1155/2011/156869 PMC3021840

[b34] Berens P. CircStat: A MATLAB toolbox for circular statistics. J Stat Softw. 31, 1–21 (2009).

