# Supplementary material for: Subliminal stimuli modulate somatosensory perception rhythmically and provide evidence for discrete perception
Source: Sci Rep. 2017 Mar 9;7:43937. doi: 10.1038/srep43937 (PMC5343432; doi:10.1038/srep43937)
Supplement: Supplementary Figures [file srep43937-s1.pdf]

Subliminal stimuli modulate somatosensory perception rhythmically and provide evidence for discrete perception

Thomas J. Baumgarten[1], Sara Königs[2], Alfons Schnitzler[1], Joachim Lange[1*]

[1]Institute of Clinical Neuroscience and Medical Psychology, Medical Faculty, Heinrich-Heine-University, 40225 Düsseldorf, Germany

[2]Department of Experimental Psychology, Faculty of Mathematics and Natural Sciences, Heinrich-Heine-University, 40225 Düsseldorf, Germany

*Corresponding author:

Joachim Lange, Institute of Clinical Neuroscience and Medical Psychology, Medical Faculty, Heinrich-Heine-University, 40225 Düsseldorf, Germany. E-mail: Joachim.Lange@med.uni-duesseldorf.de, Phone: +49 211 8113074; Fax: +49 211 8113056

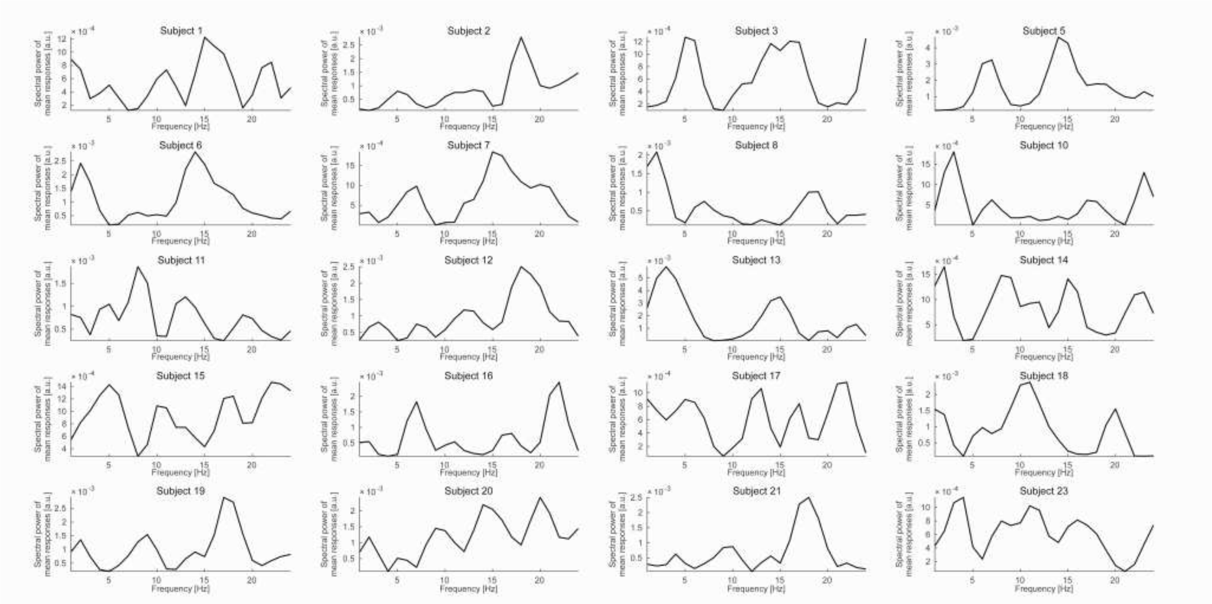

Supplementary Figure 1: Spectral decomposition of all single subject behavioral data.

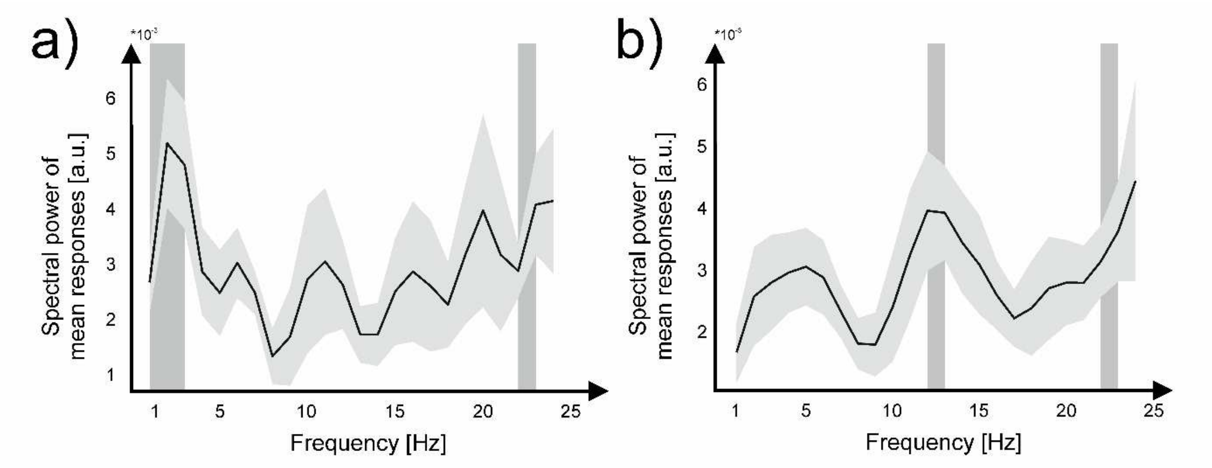

Supplementary Figure 2: Spectral decomposition of the average perceptual response rates as an effect of the time lag between subliminal stimulus and the first target stimulus. a) Spectral decomposition of the average perceptual response rates for the -50% length of the intermediate SOA condition. The grey boxes highlight frequencies with significantly increased amplitudes ($p < 0.05$, uncorrected for multiple comparisons; after correction for multiple comparisons, no significant effects remained). b) Spectral decomposition of the average perceptual response rates for the +50% length of the intermediate SOA condition. The grey boxes highlight frequencies with significantly increased amplitudes ($p < 0.05$, uncorrected for multiple comparisons; after correction for multiple comparisons, no significant effects were found). The grey shading represents the Standard Error.

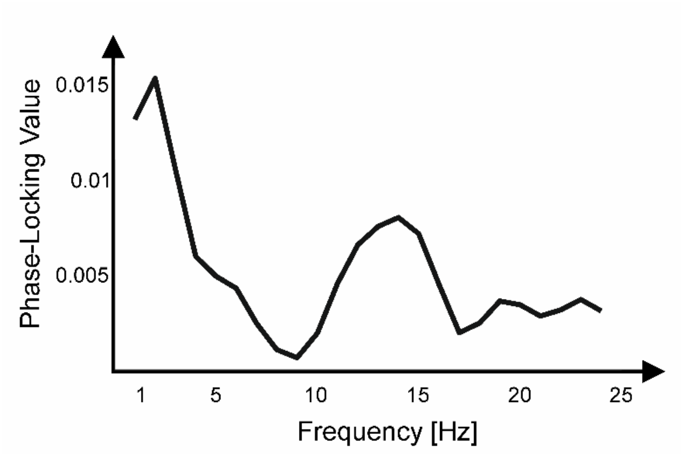

Supplementary Figure 3: Phase-locking values averaged across all subjects as a function of frequency.
